# Identification of a Novel Missense Mutation of the *PHEX* Gene in a Large Chinese Family with X-Linked Hypophosphataemia

**DOI:** 10.3389/fgene.2022.792183

**Published:** 2022-02-17

**Authors:** Yanting Yang, Yuanda Wang, Ying Shen, Mohan Liu, Siyu Dai, Xiaodong Wang, Hongqian Liu

**Affiliations:** ^1^ Department of Obstetrics and Gynecology, West China Second University Hospital, Sichuan University, Chengdu, China; ^2^ Medical Genetics Department/Prenatal Diagnostic Center, West China Second University Hospital, Sichuan University, Chengdu, China; ^3^ Key Laboratory of Birth Defects and Related Diseases of Women and Children, Ministry of Education, Sichuan University, Chengdu, China; ^4^ State Key Laboratory of Biotherapy and Cancer Center, Sichuan University, Chengdu, China; ^5^ Department of Obstetrics/Gynecology, Joint Laboratory of Reproductive Medicine (SCU-CUHK), Key Laboratory of Obstetric, Gynecologic and Pediatric Diseases and Birth Defects of Ministry of Education, West China Second University Hospital, Sichuan University, Chengdu, China

**Keywords:** X-linked hypophosphatamia (XLH), phosphate-regulating endopeptidase homolog X-linked gene (PHEX), whole-exome sequencing (WES), gene mutations, functional experiments

## Abstract

X-linked hypophosphataemia (XLH) is an X-linked dominant rare disease that refers to the most common hereditary hypophosphatemia (HH) caused by mutations in the phosphate-regulating endopeptidase homolog X-linked gene (*PHEX*; OMIM: * 300550). However, mutations that have already been reported cannot account for all cases of XLH. Extensive genetic analysis can thus be helpful for arriving at the diagnosis of XLH. Herein, we identified a novel heterozygous mutation of *PHEX* (NM_000444.5: c.1768G > A) in a large Chinese family with XLH by whole-exome sequencing (WES). In addition, the negative effect of this mutation in *PHEX* was confirmed by both bioinformatics analysis and *in vitro* experimentation. The three-dimensional protein-model analysis predicted that this mutation might impair normal zinc binding. Immunofluorescence staining, qPCR, and western blotting analysis confirmed that the mutation we detected attenuated PHEX protein expression. The heterozygous mutation of *PHEX* (NM_000444.5: c.1768G > A) identified in this study by genetic and functional experiments constitutes a novel genetic cause of XLH, but further study will be required to expand its use in clinical and molecular diagnoses of XLH.

## Introduction

Hereditary hypophosphataemia (HH) is a type of congenital disease of the phosphate-regulating homeostatic system, including phosphate-metabolism disorder and decreased renal tubular phosphate reabsorption. Thus, HH can cause rickets and osteomalacia in hypophosphataemic rickets (HR) (Carpenter, 2012). HR can be classified as FGF23-associated and non-associated, and HR is also subdivided into several forms, including X-linked hypophosphataemia (XLH), autosomal dominant hypophosphataemic rickets (ADHR), autosomal recessive hypophosphataemia (ARHR), and hereditary hypophosphataemic rickets with hypercalciuria (HHRH). Among these, X-linked hypophosphataemia (XLH) is considered the most common form of FGF23-related, inherited HR (Marcucci and Brandi, 2021; Quarles, 2012). XLH is an X-linked dominant monogenic rare disease caused by mutations in the phosphate-regulating endopeptidase homolog X-linked gene (*PHEX*; OMIM:* 300550), with an incidence of 3.9/100,000 live births and a prevalence ranging from 1.7/100,000 children to 4.8/100,000 individuals (children and adults) (Beck-Nielsen et al., 2009; Endo et al., 2015; Rafaelsen et al., 2016). The representative features of this disease are hypophosphataemia, diminished synthesis of active vitamin D (1,25 [OH]_2_ vitamin D), rickets, osteomalacia, odontomalacia, and disproportionately short stature (Haffner et al., 2019).

Individuals affected by XLH present a prominent bowing of the legs and short stature at a very early age, and up to 2/3 of children with XLH require surgical intervention (Gizard et al., 2017; Kocaoglu et al., 2011; Matsubara et al., 2008; Sharkey et al., 2015). As for affected adults, bone deformity, enthesopathy, dental abscesses, arthritis, and severe osteomalacia limit quality of life and require medical treatment for an entire lifetime (Sharkey et al., 2015). However, no studies have suggested that XLH is involved in the life expectancy of the affected individuals. The standardized treatment promotes growth, reduces bone pain, and improves dental health, eventually correcting leg deformities (Sochett et al., 2004). Early treatment augurs superior outcomes (Biosse Duplan et al., 2017; Connor et al., 2015; Mäkitie et al., 2003), and thus making appropriate early and timely diagnosis invaluable. Further, effective prenatal diagnosis of XLH is critical for optimizing a management strategy concerning affected neonates. The diagnosis of XLH depends upon genetic analysis and remains challenging (Haffner et al., 2019). There were 965 mutations of *PHEX* are listed in the Clinvar database (www.ncbi.nlm.nih.gov/clinvar), and approximately 68% of these are pathogenic or likely to be pathogenic. However, current reported mutations cannot account for all XLH cases (Lal et al., 2016; Ma et al., 2015; Zhang et al., 2019; Lin X et al., 2021), and extensive genetic analysis can therefore be helpful in achieving a diagnosis of XLH.

In the present study, we investigated a large Chinese family with XLH and detected a novel missense heterozygous mutation in the *PHEX* gene (NM_000444.5: c.1768G > A) by using whole-exome sequencing (WES), and this mutation was predicted to change glycine to serine at position 590 (p. G590S). We also substantiated the negative effect of this mutation with bioinformatics analysis and *in vitro* experimentation. Our findings broaden the spectrum of pathogenic *PHEX* mutations related to XLH and provide novel molecular evidence to allow the proper diagnosis of XLH.

## Materials and Methods

### Study Participants

The proband, a 32-year-old woman manifested XLH was enrolled at the Medical Genetics/Prenatal Diagnosis Centre of the West China Second University Hospital, Sichuan University, Chengdu, China; and we also recruited all her family members. The control group was comprised of 200 unrelated normal Han Chinese. This study was approved by the Ethical Review Board of West China Second University Hospital, Sichuan University, and informed consent was obtained from each subject or her/his guardian(s) in the case of underaged participants.

Physical and X-ray examinations were performed by specialist physicians and radiologists, respectively, and family history was acquired by doctors from the Genetic Consulting Center. Biomedical and hormonal indices were evaluated at the West China University Hospital and West China Second University Hospital of Sichuan University’s Clinical Laboratory Center. The level of electrolyte was detected by the ion-selective electrode (ISE) tests with an electrolyte analyzer (Xun-Da Medical Instrument Corporation). The level of Vit D3 and parathyroid hormone (PTH) was detected by ElectroChemiLuminescence (ECL) technology with Cobas automatic analyzer (Roche) and reagent provided by Roche. As for alkaline phosphatase (ALP), we performed the rate method by automatic chemistry analyzer (Beckman Coulter).

### Genetic Studies

WES was executed using patient DNA as follows. We collected genomic DNA from peripheral blood samples using the FitAmp Plasma/Serum DNA Isolation Kit (Epigentek Exon), implemented exon capture by the SureSelect Human All Exon V6 Kit (Agilent), and sequenced DNA through the HiSeq X system (Illumina). ANNOVAR was applied for functional annotation and the 1,000 Genomes Project, HGMD, dbSNP, and ExAC were used to filter the data.

We identified candidate pathogenic variants with respect to the patient via Sanger sequencing of the DNA from the family members as well as the normal controls. PCR reaction was performed using Golden Star T6 Super PCR Mix (TSINGKE) in a thermal cycle with an initial denaturation step of 1 min at 98°C followed by 34 cycles of 98°C for 1 min, 60°C for 15 s, and 72°C for 1 min. At the end of the thermal cycling, the reaction was a final extension at 72°C for 1 min and then immediately placed on ice. PCR reactions were amplified with the ProFlex PCR System (Thermo Fisher). We conducted sequencing of PCR products on an ABI377A DNA sequencer (Applied Biosystems). The primers of *PHEX* (NM_000444.5) we used for PCR were F, 5′ CGA​AAT​ACC​CAT​ACC​AAT​AAG​C 3′; and R, 5′ CAT​CAC​AGC​AAG​ACA​CGG​T 3'.

### Bioinformatics Analysis

To confirm the conservation of amino acid substitutions in the process of species evolution, the typical protein sequences from several different species were aligned using Clustal Omega (https://www.ebi.ac.uk/Tools/msa/clustalo/) to compare mutated positions with conserved domains. We analyzed these species: Homo sapiens (P78562), *Pan troglodytes* (H2QYE4), *Macaca mulatta* (F7HFQ1), *Canis lupus* (E2RDB0), *Bos taurus* (E1BKS5), *Mus musculus* (P70669), *Rattus norvegicus* (O35812), *Gallus* (E1BR88), *Danio rerio* (A4QP66) and *Xenopus tropicalis* (A0A6I8RG47) from Uniprot (www.uniprot.org). And we got the structure prediction of wild-type of PHEX protein from the Alphafold databased (Jumper et al., 2021; Varadi et al., 2021). The PyMOL Viewer software was used to generate the mutant PHEX protein and visualize the effects of altered residues on protein-structure models.

### Cell Culture and Immunofluorescence Staining

HeLa cells were obtained from the American Type Culture Collection (ATCC^®^). HeLa cells were grown in DMEM (Thermo Fisher) supplemented with 10% FBS (Gibco) and 1% penicillin-streptomycin (Thermo Fisher) in a humidified 5% CO2 incubator at 37°C. The expression plasmids encoding WT-*PHEX* (His-flag-tagged wild-type human *PHEX*) and mutated-*PHEX* (His-flag-tagged human mutant *PHEX* with c.1768G > A) were constructed by Vigene Biosciences company. HeLa cells were dispensed in a 6-well plastic dish containing 2 ml of fresh DMEM supplemented with 10% FBS and 1% penicillin-streptomycin at passage 3. The cells were then transfected with expressing plasmids using Lipofectamine 3,000 (Invitrogen). And we used pCMS-EGFP plasmid (Vigene Biosciences) as our positive control. For each well of a 6-well dish, 2.5 µg plasmid DNA, 5 µL of P3000 Reagent, 3.75 µL of Lipofectamine 3,000 reagent, and 250 µL of OptiMEM (Gibco) were used. After 6 h, 1 ml of fresh DMEM with 10% FBS and 1% penicillin-streptomycin was added to each well. The intense fluorescence of the GFP tag validates the transfection efficiency. Three independent transfection experiments were performed.

Cell slides with transfected cells were fixed in 4% paraformaldehyde (Sangon Biotech), permeabilized with 0.3% Triton X-100 (Beyotime), and blocked with 5% BSA (Thermo Fisher); the cell-mounted slides were then incubated with primary antibody at 4°C for 12 h. Triton and BSA dissolve in 1xPBS (Thermo Fisher). The primary antibody used was anti-Flag (1:100; ABclonal; mouse). The next day, the slides were washed three times with 1 × PBS, incubated with DyLight 594-labeled secondary antibody (1:800; Thermo Fisher; mouse) for 1 h at 25°C, and then counterstained with 4,6-diamidino-2-phenylindole (DAPI, Sigma-Aldrich) to label nuclei. Images were obtained by a laser scanning confocal microscope (Olympus FV3000). All images were captured under the same setting (brightness:0.00; contract: 0.00; gamma: 1.00). Three independent IF staining were performed.

### RNA Extraction and Quantitative Real-Time PCR

We transiently transfected expression plasmids encoding WT-*PHEX* (His-flag-tagged wild-type human *PHEX*) and mutated-*PHEX* (His-flag-tagged human mutant *PHEX* with c.1768G > A) into HeLa cells just as we mentioned previously using Lipofectamine 3,000 (Invitrogen). RNA from cultured HeLa cells was collected after 24 h of transfection. We used 1 ml Trizol (Thermo Fisher) to solubilize the cells for 5 min at room temperature and added 0.2 ml chloroform (Avantor) to promote phase separation for 2–3 min at room temperature. After centrifuging at 10,000 g for 10 min, the upper clear phase was added 0.5 ml isopropanol (Chron Chemicals) for 10 min to precipitate. We collected the precipitated RNA by centrifugation at 10,000 g for 10 min at 4°C. The precipitated RNA was re-extracted by phenol (Chron Chemicals) after resuspension. Finally, the RNA was re-precipitated with 75% ethanol (Rio et al., 2010). And the concentration and purity of the RNA were determined with a NanoDrop 2000 (Thermo Company). In our study, the OD260/OD280 of RNA showed mean values about 1.98.

We performed reverse-transcription to obtain cDNA using Hiscript III Reverse transcriptase (Vazyme), and qPCR was accomplished using Green Premix Ex Taq II (Tli RNase H Plus) (Takara Biomedical Technology) in a StepOnePlus™ Real-Time PCR System with Tower (Applied Biosystems). The PCR conditions were 40 cycles of denaturation at 95°C for 5 s and annealing at 60°C for 30 s. We analyzed results using the E−^ΔΔCT^ method, with the expression of *GAPDH* (NM_001289746.2) serving as a reference gene. Each reaction was repeated three times. The primers for quantitative real-time PCR (qPCR) of *PHEX* (NM_000444.5) were as follows: F, 5′ GAA​GCC​TTT​CTT​TTG​GGG​A 3′; and R, 5′ ATG​CCT​CTG​TTC​ATC​GTG​G 3′. And the primers of *GAPDH* (NM_001289746.2) were as follows: F, 5′ ACG​GAT​TTG​GTC​GTA​TTG​GG 3′; and R, 5′ CGC​TCC​TGG​AAG​ATG​GTG​AT 3′. The efficiency of amplification curves was analyzed using LinRegPCR software. Three independent qPCR assays were performed.

### Western Blotting Analysis

We transiently transfected expression plasmids encoding WT-*PHEX* (His-flag-tagged wild-type human *PHEX*) and mutated-*PHEX* (His-flag-tagged human mutant *PHEX* with c.1768G > A) into HeLa cells as we mentioned previously by Lipofectamine 3,000 (Invitrogen). Proteins in the cultured HeLa cells were extracted using a universal protein extraction lysis buffer (Bioteke) containing a protease-inhibitor cocktail (Roche). After 30 min of lysis, we performed a centrifuge at 10000 g for 5 min and collected the clear upper layer. Denatured proteins were separated electrophoretically on 10% SDS-PAGE Gel (Epizyme) and transferred to a polyvinylidene difluoride (PVDF) membrane (Millipore) for immunoblot analysis. The primary antibodies that we used were anti-Flag (1:1,000, Abcam; mouse) and anti-GAPDH (1:5,000, Abcam; rabbit). Three independent western blotting analysis were performed.

### Statistical Analysis

Statistical analyses were conducted using SPSS 17.0 software. All the data of biomedical and hormonal indices were presented as the means ± SD. Statistical significance between two groups was calculated using a nonparametric test. The level of significance was set at *p* < 0.05. And we used SEM as the error bars.

## Results

### Characteristics of the Clinical Phenotype

**FIGURE 1:**
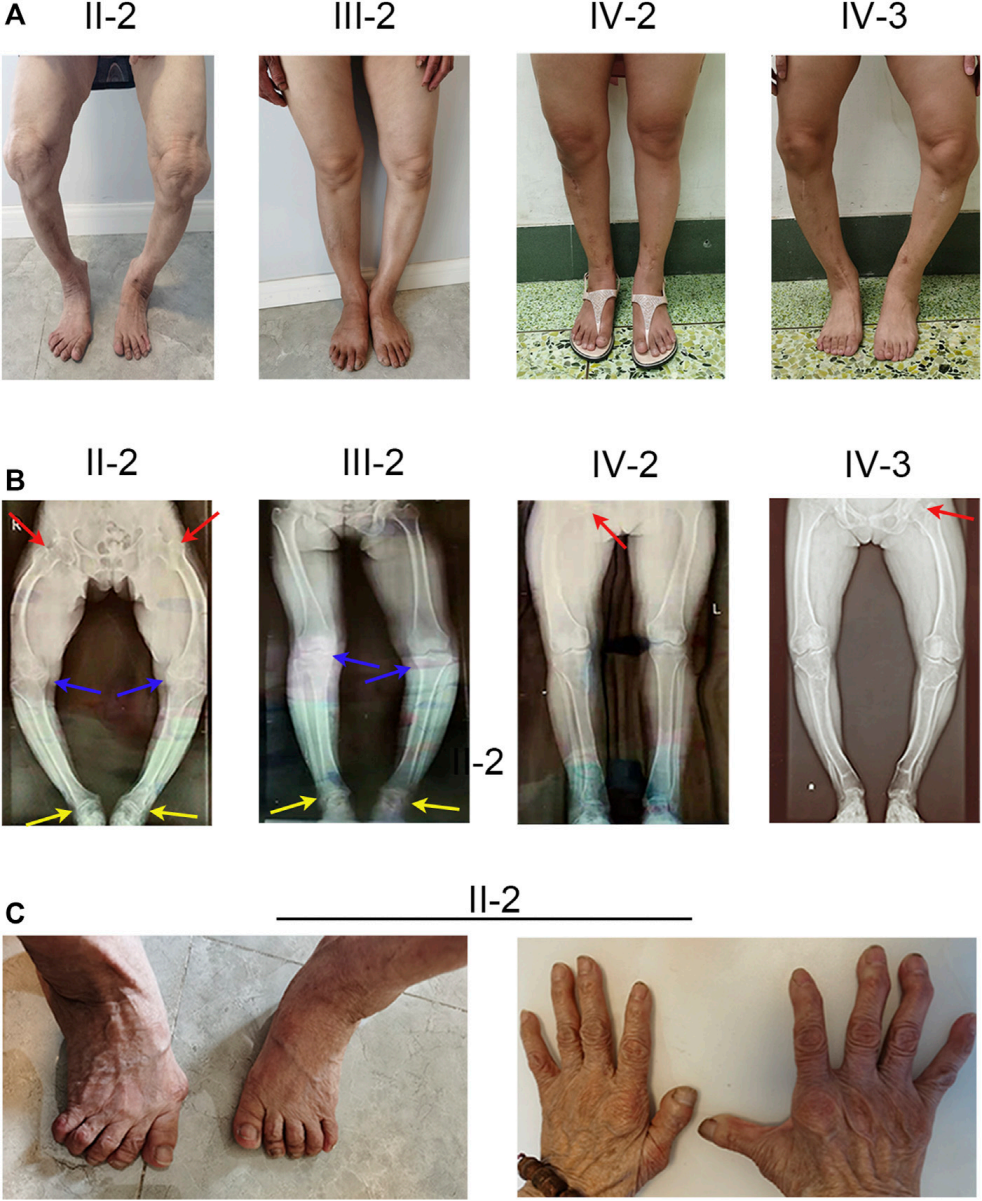
Phenotype and X-ray autoradiographs of the family. **(A)** Clinical phenotype of the affected individuals in the family. The patients present with short stature and obviously bowed legs. **(B)** The radiographs of the lower limbs of the patients show varying degrees of genu varum and osteoarthritis, with osteophytes on the joint margins (indicated by arrows. hips, red; keens, blue; ankles, yellow). **(C)** Patient II-2 exhibits severe joint deformity of both hands and feet due to the lack of earlier treatment.

The proband (IV-2) presented to our hospital with short height (143 cm), genu varum, a waddling gait, and obvious family history. The proband’s grandmother (II-2), mother (III-2), and elder sister (IV-3) also exhibited these same phenotypes. The proband (IV-2) had already given birth to a girl (V-1) who showed leg bowing at a very early stage. And the girl (V-1) died of an unknown cause at 1 year old. The proband’s grandmother (II-2) was 80 at the time and showed unexplained rickets during childhood, without receiving any medication. Patient II-2’s height was 125 cm, with obvious bowing of her legs. In her young adult years, she performed simple farm work but demonstrated a waddling gait. She gradually manifested difficulty in walking and progressive bone pain. And due to a lack of appropriate treatment, the joints of both her hands and feet showed obvious deformities (Figure 1C). The proband’s mother (III-2) was at the time 55 years of age and 152 cm tall, and similar to patient II-2 exhibited short stature, genu varum, and difficulty moving; but received oral calcium supplementation at approximately 40 years of age. She can still perform simple tasks with occasional bone pain. The proband’s elder sister (IV-3) who achieved a height of 137 cm also showed the aforementioned signs and bore a boy (V-2) who shows no signs of rickets or osteomalacia. Of note, with advances in medicine, both the proband and patient IV-3 received not only oral treatment but also surgical limb correction (tibial osteotomy) during puberty; however, patient IV-3 still shows overt leg bowing (Figure 1A). Compared with patient IV-3, the proband manifests a more favorable lower-limb appearance, but still shows a waddling gait (Figure 1A). None of our patients showed specific syndromic facial features, and none exhibited dental diseases or complained of recurrent fractures.

To investigate the cause of their symptoms, the patients underwent X-ray examinations. The results revealed short stature with varying degrees of genu varum (Figure 1B). The radiographs also showed varying degrees of osteoarthritis—with the osteophytes on the joint margins included in the hips, knees, and ankles. These results are consistent with typical radiographic features in adults with XLH (Figure 1B). Due to the lack of early treatment, patient II-2’s radiograph shows severe osteoarthritis in both hips, knees, and ankles, with obviously narrowed articular cavities (Figure 1B). Biochemical tests depicted a low level of serum phosphate in all patients, and in patient II-2, patient III-2, and in the proband (VI-2), this was combined with elevated alkaline phosphatase (ALP); the level of serum parathyroid hormone (PTH) was concomitantly above normal in all patients (Table 1). These results were consistent with the diagnosis of XLH. In contrast to rickets, which was secondary to vitamin D or calcium deficiency, all the patients exhibited normal concentrations of calcium and vitamin D3 (Table 1). Based on these findings, these patients were diagnosed with XLH. However, we nevertheless recommend genetic analysis, particularly with respect to a mutation in *PHEX* (Haffner et al., 2019).

**TABLE 1:** Clinical and biochemical features of the family under study.

Patient	II-2	III-2	IV-2	IV-3	Reference range
Gender	F	F	F	F	—
Age(y)	78	55	26	33	—
Height (cm)	125	151	143	137	—
Phosphate (mmol/L)	0.681 ± 0.002	0.73 ± 0.004	0.63 ± 0.003	0.72 ± 0.004	0.085–1.51 mmol/L
Calcium (mmol/L)	2.292 ± 0.068	2.183 ± 0.101	2.329 ± 0.135	2.217 ± 0.105	2.11–2.52 mmol/L
Vit D3 (ng/ml)	8.067 ± 0.569	13.793±	16.797 ± 0.166	8.730 ± 0.115	30–100 ng/ml
ALP (U/L)	173.320 ± 5.457	118.10 ± 8.805	120.013 ± 8.993	93.857 ± 6.692	35–100 mmol/L
PTH (ng/L)	50.579 ± 2.384	18.093 ± 1.500	11.08 ± 1.037	14.836 ± 0.646	1.60–6.90 pmol/L

ALP, alkaline phosphatase; PTH, parathyroid hormone.

### Identification of a Heterozygous c.1768G > A Mutation of *PHEX* in Patients With X-Linked Hypophosphataemia

**FIGURE 2:**
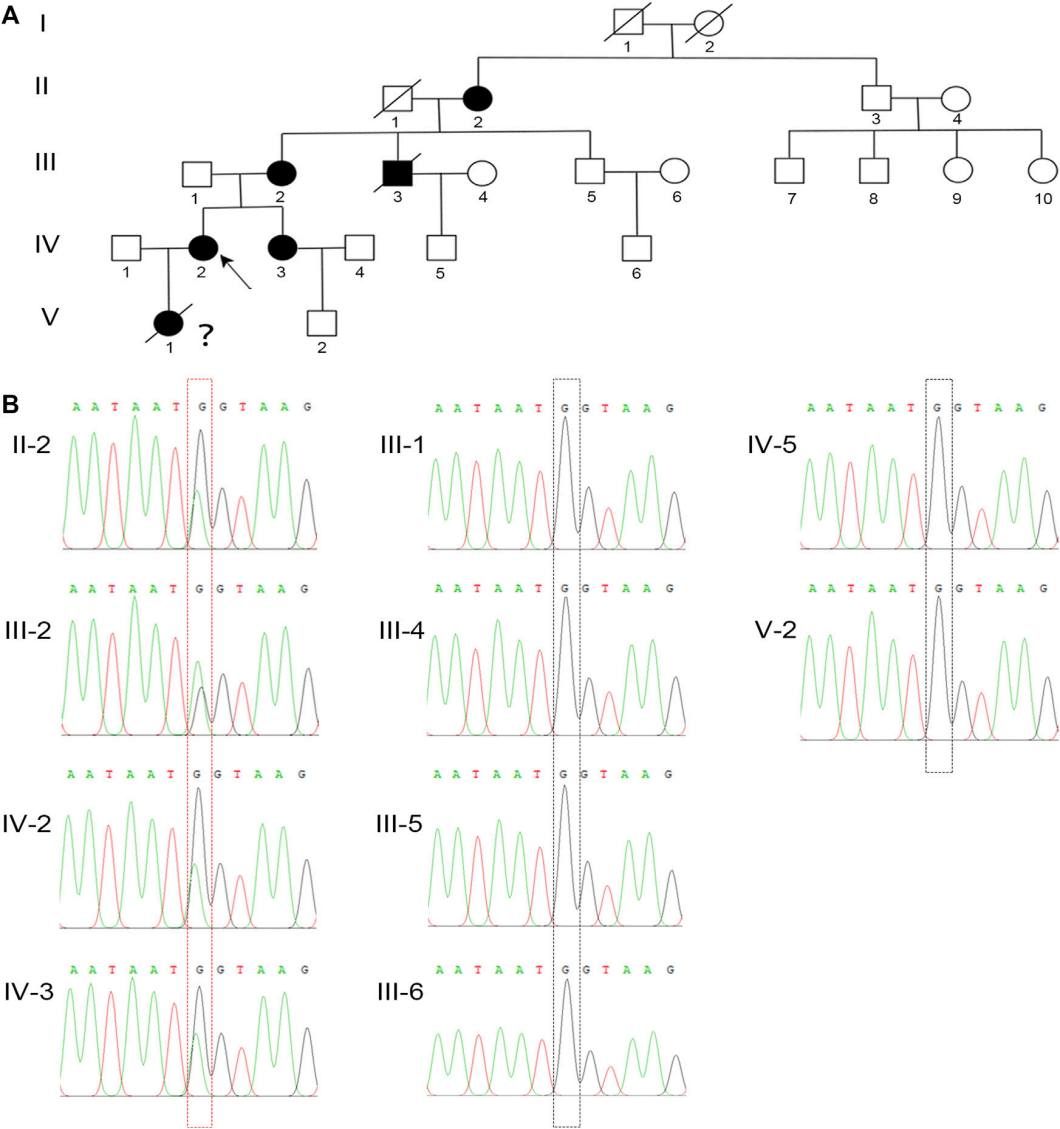
A novel missense mutation in *PHEX* was detected in the XLH family. **(A)** The family pedigree of this XLH family. The black circles and square show the affected individuals with XLH, and the proband is indicated by the black arrow. Because of the lack of clinical and genetic analyses, the diagnosis and genotype of V-1 are uncertain, which is indicated by a question mark. **(B)** Sanger sequencing confirmed a heterozygous G-to-A transversion at nucleotide c.1768 (red dotted box) of the *PHEX* gene in all XLH patients in this family, while the unaffected individuals were identified as wild type (black dotted box).

To elucidate the genetic cause of HH in this family, we performed WES on all affected individuals. We removed variants if the following conditions were met: (a) the minor allele frequency was greater than or equal to 1% in ExAC Browser, gnomAD, or the 1,000 Genome Projects—considering that pathogenic variants that cause XLH are rare in humans; (b) the variant was not predicted to be deleterious by SIFT, PolyPhen-2, or MutationTaster tools; and (c) the variant was in noncoding exons, 3′- or 5′-untranslated regions, or intronic sequences—except for canonical splice sites. Surprisingly, a heterozygous mutation in *PHEX* (NM_000444.5: c.1768G > A) was screened, which is the known causative gene for XLH. To confirm the mutation’s distribution in this family, we assessed this mutation in all family members by Sanger sequencing, including four patients and six unaffected members (Figure 2A). All patients harbored this mutation, while other members were identified as wild type (Figure 2B). Furthermore, we did not find this mutation in 200 normal controls, which also supported that this mutation might be the genetic cause of this family.

### The Heterozygous c.1768G > A Mutation of *PHEX* Impairs Its Expression

**FIGURE 3:**
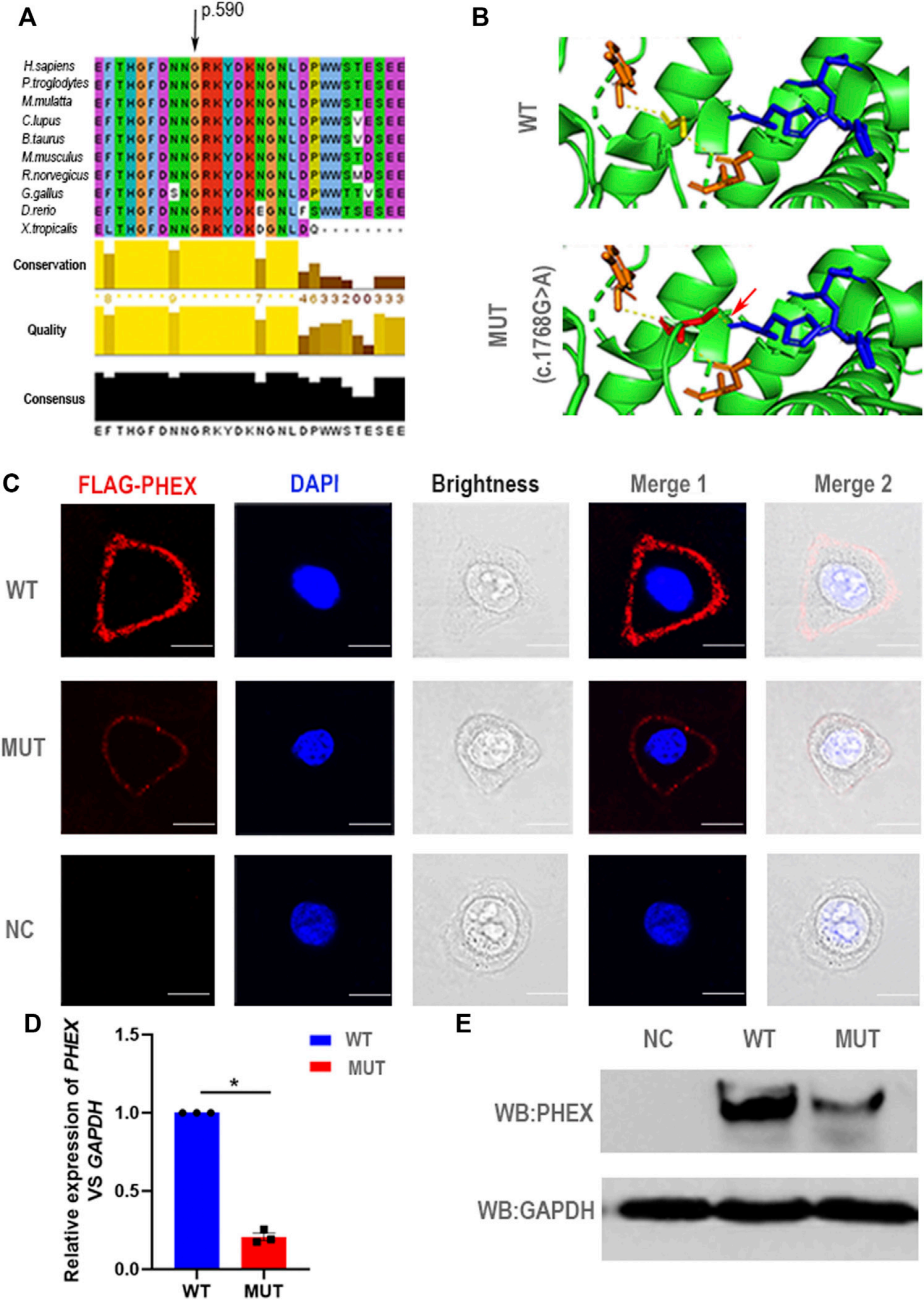
Negative effect of the novel mutation in *PHE*X. **(A)** The mutant position we detected is highly conserved among many species. **(B)** Structural illustration of the missense mutation in *PHEX*. The mutation site in the wild-type PHEX protein model (WT) and mutant PHEX protein model (MUT) are shown as sticks and highlighted in yellow and red. The interrelated putative zinc-binding sites are also represented by sticks and colored in blue. The polar contacts of the target residues are shown as yellow dashed lines, and relative amino acids are shown as orange sticks. In the wild-type protein, Gly590 forms two polar contacts predicted by PyMOL; while in the mutant protein, Ser590 forms three polar contacts due to the addition of one polar contact with His584 that was deemed to be a zinc-binding site predicted by PyMOL. **(C)** The immunofluorescent distribution of PHEX protein. The decline expression level of PHEX protein was noted using immunofluorescence staining of HeLa cells transfected with mutant-*PHEX* plasmid compared to cells transfected with WT-*PHEX* plasmid (red, FLAG-PHEX; blue, DAPI). Scale bars represent 10 μm. Three independent experiments were performed. **(D)** Using qPCR, we observed that *PHEX* mRNA expression levels in the cultured HeLa cells transfected with mutant-*PHEX* plasmid were sharply attenuated compared to the cells transfected with WT-*PHEX* plasmid. (nonparametric test; *p < 0.05; error bars, s.e.m.). Three independent experiments were performed. **(E)** The western blotting results showed that PHEX protein was scarcely detectable in HeLa cells transfected with mutant-*PHEX* plasmid compared with the cells transfected with WT-*PHEX* plasmid. Three independent experiments were performed.

For a deeper appreciation of the mutation that we identified in *PHEX*, we performed a relative bioinformatics analysis. Protein-conservation analysis first showed that position 590 was highly conserved among many species (Figure 3A), suggesting that this region might be vital for protein function. Based on the structure prediction of wild-type PHEX from the Alphafold database (Jumper et al., 2021; Varadi et al., 2021), we generated three-dimensional protein models of the mutant protein. This single nucleotide mutation was predicted to change the glycine to serine at position 590. Importantly, the glycine in the wild-type protein was predicted to form two polar contacts, whereas the changed serine in the variant was predicted to form three contacts with surrounding residues—adding one with histidine (p.584) (a putative zinc-binding site (Rowe et al., 2005)) (Figure 3B). We assumed that the altered polar contact of the putative zinc-binding site may thus lead to abnormal zinc binding.

To further elucidate the damaging effects of *PHEX* mutation on its expression, the WT-*PHEX* (His-flag-tagged wild-type human *PHEX*) and mutated-*PHEX* (His-flag-tagged human mutant *PHEX* with c.1768G > A) plasmids were transiently transfected into HeLa cells respectively. Analysis of immunofluorescence staining showed that PHEX was visibly expressed in the plasma membrane of HeLa cells transfected with WT-*PHEX* plasmid, while PHEX staining was only marginally detectable in the plasma membrane of cells that overexpressed mutated-*PHEX* plasmid (Figure 3C). To investigate this mutation affection in detail, we transfected plasmid in HeLa cells and collected RNA. Through qPCR detection, our results show a sharply attenuated mRNA level of the mutation compared to the wild type (Figure 3D). We also demonstrated the mutation’s potential deleteriousness by using western blotting analysis and uncovered consistently reduced expression of the protein (Figure 3E).

Thus, the novel heterozygous mutation in *PHEX* (NM_000444.5: c.1768G > A) causing the impaired function of PHEX protein might be associated with the abnormal protein structure of zinc binding and the decreased transcription level. These data together strongly suggest that the novel heterozygous mutation c.1768G > A in *PHEX* might be the genetic cause of XLH in this family.

## Discussion

XLH—an X-linked dominant monogenic disorder—is the most common form of hereditary hypophosphataemia, representing approximately 80% of all hypophosphataemic rickets (Guven et al., 2017; Holm et al., 2001; Ruppe et al., 2011). In our study, we identified a novel heterozygous mutation in *PHEX* (NM_000444.6 c.1768G > A) in a large Chinese family. This missense mutation was predicted to cause an abnormal polar contact with a putative zinc-binding site, thus might cause abnormal zinc binding by PHEX protein (Jumper et al., 2021; Varadi et al., 2021). Meanwhile, western blotting showed that this novel mutation resulted in the diminished expression of PHEX. Therefore, we suggested that this heterozygous mutation we found in *PHEX* (NM_000444.6 c.1768G > A) might be the genetic cause of XLH in this family.

In previous studies, 50% of the missense mutations in the *PHEX* have been suggested to be associated with protein trafficking/localization (Sabbagh et al., 2003). Some researchers supposed that these missense mutations cause misfolding and retention of the mutant PHEX protein in the endoplasmic reticulum (ER) resulting from the unsuccessful PHEX protein trafficking/localization. In our study, we also found a novel missense mutation in *PHEX* (NM_000444.6 c.1768G > A). However, we detected this missense mutation could reduce the PHEX expression, and did not change the localization of PHEX protein in HeLa cells. According to the mutant three-dimensional protein model predicted by PyMOL, this mutation causes an abnormal polar contact with a putative zinc-binding site without misfolding and retention (Jumper et al., 2021; Varadi et al., 2021). While the mechanism needs more study in the future.

In 1995, a phosphate-regulating gene with sequence identity like endopeptidases (*PHEX*) was for the first time described as the direct genetic cause of XLH (The HYP Consortium 1995). *PHEX* encodes a cell-surface-bound protein-cleavage enzyme PHEX that consists of a short amino terminal cytoplasmic domain, a single transmembrane domain, and a large extracellular domain containing a zinc-binding motif and conserved cysteine residues (Sabbagh et al., 2001). Due to a sequence identity similar to that of the neutral endopeptidases, PHEX is regarded as a regulating factor in phosphate homeostasis (Amatschek et al., 2010). The deletion of *PHEX* contributed to the enhanced secretion of fibroblast growth factor 23 (FGF23) in mice (Hyp mouse), thus occupying a critical role in the complicated secretory network, further affecting the regulation of systemic phosphate homeostasis and vitamin D metabolism (Itoh and Ornitz, 2008; Liu Z et al., 2006). The excessive level of FGF23 noted in hypophosphataemia was then shown to suppress 1,25-dihydroxyvitamin D levels and induce rickets or osteomalacia in humans and mice (Bai et al., 2004; Fukumoto and Yamashita., 2002; Shimada et al., 2001,^2005^).

Inactivating mutations of *PHEX* results in increased circulating and intact FGF23, which ultimately causes HH disorders and defections in bone mineralization in humans and other mammals (Bai et al., 2016; Liu T et al., 2006; Murali et al., 2016; Quarles, 2008; Quarles, 2012; Yuan et al., 2008). Unfortunately, because of the situation of COVID-19, our patients reject to come to our hospital again. We didn’t get complete the FGF-23 levels in our patients. But functions of FGF23 have been elaborated clearly, with studies illustrating the resorptive regulation of phosphate and the production as well as catabolism of 1,25-dihydroxyvitamin D and the expression of α-Klotho; the latter is an anti-aging hormone that principally acts on the kidney (Liu T et al., 2006; Quarles, 2008; Quarles, 2012; Yuan et al., 2008). Although these functions reflect the plethora of manifestations of XLH, the molecular mechanisms underlying the mutations in *PHEX* that lead to the elevated secretion of FGF23 and the inherent functions of PHEX are still unclear. PHEX is hypothesized to be the protease responsible for the cleavage and corresponding inactivation of FGF23 due to its high sequence identity relative to other endopeptidases. And while some investigators have shown that FGF-23 is a PHEX substrate (Bowe et al., 2001; Campos et al., 2003), others suggest that FGF23 is not a direct PHEX substrate and cannot demonstrate PHEX-dependent cleavage of FGF-23 *in vitro* (Guo et al., 2001; Liu et al., 2003; Quarles and Drezner, 2001). In fact, an animal study indicated that *Phex* mutations led to elevated FGF-23 levels due to increased transcription of the *Fgf23* gene in osteoblasts and osteocytes (Quarles and Drezner, 2001). Thus, one hypothesis posits the existence of an intermediate pathway between PHEX and FGF-23 that augments FGF-23 levels, leading to phosphaturia and hypophosphataemia; but this assertion requires further study.

Besides, the diagnosis of XLH remains challenging. Previous reports show that XLH, ADHR, and ARHR possess similar clinical features and even reflect similar biochemical characteristics. While the genetic causes of XLH (*PHEX*), ADHR (*FGF23*), and ARHR (*DMP1* and *ENPP1*) have already been clearly described, there remain vast differences in their respective inheritance patterns and underlying pathogenesis (Liu T et al., 2006; Quarles, 2008; Quarles, 2012; Rafaelsen et al., 2016; Yuan et al., 2008). In addition, the physical manifestations may also be misdiagnosed as metaphyseal dysplasia, nutritional rickets, or physiological bowing (Carpenter et al., 2011). Therefore, it is necessary to clinically differentiate the disease cause before commencing treatment and genetic counseling, and genetic analysis is essential for the proper diagnosis of XLH (Saito et al., 2009). As soon as the diagnosis is established, it is recommended to initiate a combination of oral phosphorus (phosphate salts) and active vitamin D (calcitriol or alfacalcidol) for the treatment of children with XLH (Haffner et al., 2019). For symptomatic adults, the clinical recommendation is also the combination of oral treatment to reduce the incidence of osteomalacia and its consequences, and to improve dental health (Haffner et al., 2019); as increased calciuria and even nephrocalcinosis are reported in 30–70% of patients undergoing conventional treatment (Eddy et al., 1997; Goodyer et al., 1987; Keskin et al., 2015). Examples of conventional treatment are the orthopedic procedures usually used to correct deformities and to treat pathological fractures; and surgery is still associated with a high risk for recurrence of limb deformities, particularly prior to puberty (Gizard et al., 2017). Based upon the adverse effects tied to current treatment—and although favorable outcomes can result from appropriate early intervention—affected patients still confront clinical problems even with formal treatment. Thus, timely diagnosis and prenatal diagnosis are extremely valuable.

## Conclusions

In summary, although the specific mechanisms underlying mutations in *PHEX* that resulted in XLH still require further exploration, our findings expand the genetic and molecular evidence with respect to proper clinical and prenatal diagnosis of XLH. We also acknowledge that genetic analysis can play a critical role in XLH diagnosis and prognosis, thus providing additional beneficial knowledge related to genetic counseling.

## Data Availability

The datasets for this article are not publicly available due to concerns regarding participant/patient anonymity. Requests to access the datasets should be directed to the corresponding authors.
